# Restoring Coastal Plants to Improve Global Carbon Storage: Reaping What We Sow

**DOI:** 10.1371/journal.pone.0018311

**Published:** 2011-03-29

**Authors:** Andrew D. Irving, Sean D. Connell, Bayden D. Russell

**Affiliations:** Southern Seas Ecology Laboratories, School of Earth and Environmental Sciences, The University of Adelaide, Adelaide, South Australia, Australia; University of Zurich, Switzerland

## Abstract

Long-term carbon capture and storage (CCS) is currently considered a viable strategy for mitigating rising levels of atmospheric CO_2_ and associated impacts of global climate change. Until recently, the significant below-ground CCS capacity of coastal vegetation such as seagrasses, salt marshes, and mangroves has largely gone unrecognized in models of global carbon transfer. However, this reservoir of natural, free, and sustainable carbon storage potential is increasingly jeopardized by alarming trends in coastal habitat loss, totalling 30–50% of global abundance over the last century alone. Human intervention to restore lost habitats is a potentially powerful solution to improve natural rates of global CCS, but data suggest this approach is unlikely to substantially improve long-term CCS unless current restoration efforts are increased to an industrial scale. Failure to do so raises the question of whether resources currently used for expensive and time-consuming restoration projects would be more wisely invested in arresting further habitat loss and encouraging natural recovery.

## Introduction

As the varied consequences of a changing climate continue to challenge our technical capacity [1] and political will [2] to globally stabilize and manage greenhouse gas emissions, numerous potential solutions have gained traction among the scientific community [3] and general public [4]. Among these is the industrial-scale artificial capture and long-term storage of anthropogenic CO_2_ before it is released to the atmosphere and contributes to the greenhouse effect. We already possess the technical expertise to engineer such feats by pumping liquefied CO_2_ into porous geological formations more than 1 km underground [5]. However, uncertainty remains over the considerable expense and potentially damaging side-effects of such operations, including unforeseen geological de-stabilisation and chronic CO_2_ leakage into marine and terrestrial environments, and ultimately into the atmosphere [6]. Additionally, routine use of such procedures will be unlikely until at least 2025 [7].

Compared to the attention given to methods of artificial carbon capture and storage (CCS), natural carbon sinks such as terrestrial and aquatic vegetation have often been overlooked or considered supplementary for management [8]. This disparity probably stems from the quantified inability of such biological reservoirs to compensate for the sheer volume of anthropogenic carbon currently produced (∼440×10^6^ vs ∼8500×10^6^ t C yr^−1^, respectively; [9], [10]). Nevertheless, natural means of CCS are immediately available, cost-effective, publicly supported, and offer many complementary benefits such as the preservation of biodiversity and other natural resources. When coupled with current uncertainties regarding artificial CCS techniques, natural approaches appear to warrant serious consideration as an important contributor to managing the carbon problem.

**Figure 1:**
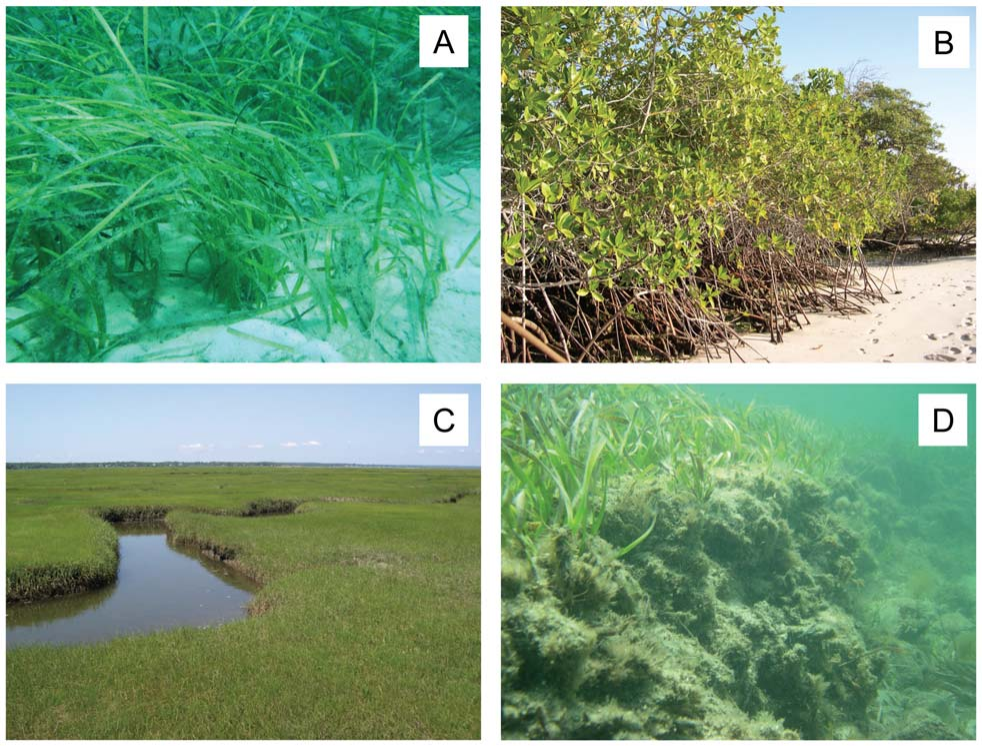
Major carbon-storing habitats on tropical and temperate coasts. Degradation and loss of (A) seagrass meadows, (B) mangrove forests, and (C) salt marshes may release hundreds to thousands of years worth of stored carbon through exposure and breakdown of below-ground biomass, shown in (D) for seagrasses. Photo credits: Andrew Irving.

As a carbon sink, the ocean can absorb up to one-third of anthropogenic CO_2_ in the atmosphere [11]. It therefore seems fortunate that coastal vegetation such as seagrasses, mangroves, and salt marshes (Fig. 1A–C) capture and store carbon at non-trivial amounts of 60–210 t C km^−2^ yr^−1^ [9], [12], and do so with far greater efficiency than their terrestrial counterparts (e.g. tropical forests only store 2.3–2.5 t C km^−2^ yr^−1^; [9]). Under favorable conditions, the majority of captured carbon may be stored as below-ground biomass (e.g. peat) for decades to possibly thousands of years.

Like most biological resources, however, coastal vegetation has undergone extensive declines in global distribution and abundance [13], culminating in the loss of ∼ one-third of the world's seagrass meadows and mangrove forests, and more than one-half of salt marshes, during the past century [14], [15], [16]. Losses have been, and continue to be, largely driven by anthropogenic stressors, including pollution (e.g. eutrophication, turbidity), altered sedimentation regimes, and direct physical disturbance (e.g. reclaiming coasts). To date, losses are thought to have reduced global CCS rates by at least 25% [13], and continued losses may exacerbate the carbon problem by exposing below-ground biomass that can release hundreds to thousands of years worth of stored carbon as it erodes and degrades (Fig. 1D). Precise numbers on the potential magnitude of such ‘re-activation’ of stored carbon are scarce, yet Cebrian [17] conservatively estimated that the loss of the world's mangrove forests to date has resulted in the release of 3.9×10^8^ tonnes of previously stored carbon.

Given the enormity of the carbon problem, anything less than a thorough consideration of all possible methods of mitigation could appear neglectful. Thus, it is worth asking whether trends of coastal habitat loss can be reversed to increase global CCS. If future losses of habitat can be prevented such that they do not occur at the expense of any gains, which is a significant challenge given the magnitude of global exploitation of coastal environments [18], facilitating natural recovery and expansion of habitats may be a critical first step. However, many species of coastal plants require decades to centuries to recover from disturbance because they depend primarily on clonal expansion rather than sexual reproduction for population growth [19]. Therefore, direct intervention through habitat restoration may represent a way to more rapidly improve rates of natural CCS. Habitat restoration is a potentially powerful approach, but the task of re-creating complex ecosystems presents many challenges that so far have typically produced viable self-sustaining populations well below parity of effort (e.g. 35–50% successful establishment of planted seagrass units [20]) and have generally confined restoration projects to small spatial scales (≤1 ha: [21]).

The purpose of this study was twofold. Firstly, we quantified the decline in global rates of CCS by mangroves, seagrasses, and salt marshes due to their historical decline in abundance. Based on this information, we (secondly) compared how rates of CCS might improve under future scenarios of habitat recovery and restoration. Of particular interest was to compare the long-term benefits of using different intensities of habitat restoration to provide some indication of the effort needed to produce a sizeable effect. While we recognize that restoration of habitats provides numerous benefits additional to CCS (e.g. nutrient cycling, coastal stabilization, preservation of biodiversity), we focus on CCS as a current topic of significant global concern and debate. Given the considerable amounts of time and expense involved in most restoration programs (e.g. seagrass averages US$48,700 ha^−1^: [20], adjusted to 2010 dollar value), coupled with their often modest chances of success (35–50%: [20]), it is debatable whether the investment of finite resources would instead be better directed toward approaches that limit further habitat loss and promote natural recovery.

## Materials and Methods

Data used to quantify changes to global rates of CCS by coastal vegetation were sourced from literature describing historical habitat abundance and/or calculated rates of CCS. We focused on seagrasses, mangroves, and salt marshes because they are well-known for their capacity to store significant amounts of carbon for long periods [8], [13], and also because of their near-global presence on tropical and temperate coasts. We note that forests of kelp and other macroalgae also constitute a major coastal habitat, particularly at temperate latitudes, but they are excluded from this study because they are essentially ephemeral in their CCS capacity; i.e., carbon captured in their tissue is released as the plant decays or dies, with none of it stored below-ground since the plants possess no root structure.

For each habitat, global CCS was calculated by multiplying estimates of global habitat area, often averaged across several data sources, by quantified rates of CCS per unit area. Few estimates of CCS rates for each habitat are available in the literature, and so we used the highest and lowest rates we could find to provide some indication of variance in historical decline of CCS. While this is a relatively simple method that does not include compounding influences such as variation among species and latitudes, it nonetheless provides similar estimates to studies using more complex methods (e.g. [13]), and forms a standard baseline for relative predictions of increasing CCS under different scenarios of future habitat recovery. Where possible, our calculated global CCS was partitioned into decadal increments (i.e. the annual rate of global CCS by that habitat in each decade) because of instances of rapid habitat decline in particular decades (see Results). However, sparse data describing habitat abundance sometimes limited this approach, particularly in earlier years. Additionally, data describing changes in CCS by salt marshes were restricted to the continental USA because poor estimates of salt marsh abundance elsewhere precluded calculations of their global abundance. Even so, patterns observed in the USA are likely to be representative of many locales since salt marshes are one of the most common habitats “reclaimed” during coastal development [14]. Table 1 provides a summary of key values and data sources used in calculations.

**Table 1:** Summary of key data sources and values used in calculations of global historical CCS rates and future changes under different habitat recovery and restoration scenarios.

(a) Historical global habitat abundance ×10^3^ km^2^	Time interval	Seagrass (16)	Mangrove (12, 15)	Salt Marsh (USA only) (14, 34)
	1879–1930	174.75		
	1930–1940	174.84		7721.609
	1940–1950	174.57		7296.080
	1950–1960	172.64		6474.491
	1960–1970	173.04		5257.010
	1970–1980	170.51	36957.64	4366.717
	1980–1990	126.02	30567.51	3629.156
	1990–2000	128.19	24177.38	
	2000–2006	125.54		

(b) Quantified CCS rates:t C km^−2^ yr^−1^	Seagrass	Mangrove	Salt Marsh	
	83 (9)	139 (9)	210 (9)	
	83 (13)	139 (13)	151 (13)	
	133 (8)	60 (12)		

(c) Seagrass restoration	Area planted (km^2^, USA only)	Average restoration success rate	Expected area restored (km^2^)	Potential global area restored* (km^2^)
	0.78 (20)	42% (20)	0.33	4.59

*Potential global area restored is based on extrapolating the amount of successful seagrass restoration in the USA, using the relative proportion of the world's seagrass contained within the USA (∼7.1%). Given restoration efforts in the USA are likely greater than many other countries, this may over-estimate current global restoration effort.

Data sources are listed in parentheses.

Forecasting improvements to global CCS under different scenarios of habitat recovery and restoration was done by multiplying quantified rates of CCS per unit area by estimates of global habitat area resulting from either natural recovery alone, or natural recovery combined with restoration. Initially, it was hoped that improvements could be calculated for all three habitats, yet literature searches soon revealed that calculations could only be reliably done for seagrasses because good estimates of global rates of seagrass expansion (taken as indicative of recovery), as well as a key synthesis of rehabilitation efforts [20], were available. While results are therefore focused on seagrasses, similar patterns are likely for mangroves and salt marshes, though probably over different time scales (e.g. mangroves are slower growing and therefore may take longer to recover).

The rate of natural seagrass recovery was based on global measures of seagrass expansion presented in Table 1 of [16]. Note that seagrass is in global decline because the overall rate of expansion is overwhelmed by the rate of loss, yet some locations exhibit greater rates of expansion than loss, which can be used to calculate a nominal rate of recovery for forecasts described herein. Over a 70-year period, [16] quantified 879 km^2^ of seagrass expansion (against losses exceeding 9,000 km^2^) across study areas totaling 11,592 km^2^ around the world. These measures equate to an average global seagrass expansion rate of 1.08% per decade. This value may be an underestimate since small-scale studies have shown rates of recovery up to 7.5% yr^−1^ [22]. Indeed, recovery is likely to be greater that 1.08% per decade under favourable water quality and physical conditions, but given the uncertainty regarding favourable future coastal conditions [23] such values are yet to be reliably determined. Furthermore, we use the value of 1.08% recovery per decade because this estimate is based on the truly global synthesis of [16], which provides necessary parity for the global scale calculations described herein.

The total global seagrass restoration effort has never been fully quantified, but a comprehensive synthesis by [20] tallied a total of 0.78 km^2^ for the USA since the 1960s. Assuming an average restoration success rate of 42% (after [20]), one can expect the establishment of ∼0.33 km^2^ of seagrass from the 0.78 km^2^ planted. Extrapolating efforts in the USA (supporting 7.1% of the world's seagrass: [24]) to the remainder of the world would give a successful global seagrass restoration effort of 4.59 km^2^. In other words, if the restoration efforts of the USA were replicated throughout the world, 4.59 km^2^ of seagrass would have been restored globally. This is certainly an overestimate because the USA has a long history of seagrass restoration relative to many other countries, but it nevertheless provides a quantifiable benchmark for future restoration efforts.

For the current study, future changes to global CCS by seagrass meadows were calculated under a ‘recovery only’ scenario, where seagrasses were allowed to recover at a rate of 1.08% per decade from 2010 to 2100. Results were then compared to CCS rates when this level of recovery was combined with seagrass restoration at i) the current effort per decade (i.e. recovery + restoration of 4.59 km^2^ per decade), ii) 10-times the current effort per decade (i.e. recovery+45.94 km^2^ per decade), and iii) 100-times the current effort per decade (i.e. recovery+459.4 km^2^ per decade). Finally, these forecasts were compared to a ‘continued decline’ scenario where loss of seagrass persists at the 1980–2000 average rate of 0.02% per decade (calculated from Table S2 in [16]).

## Results

### Historical habitat losses and decline in CCS

**Figure 2:**
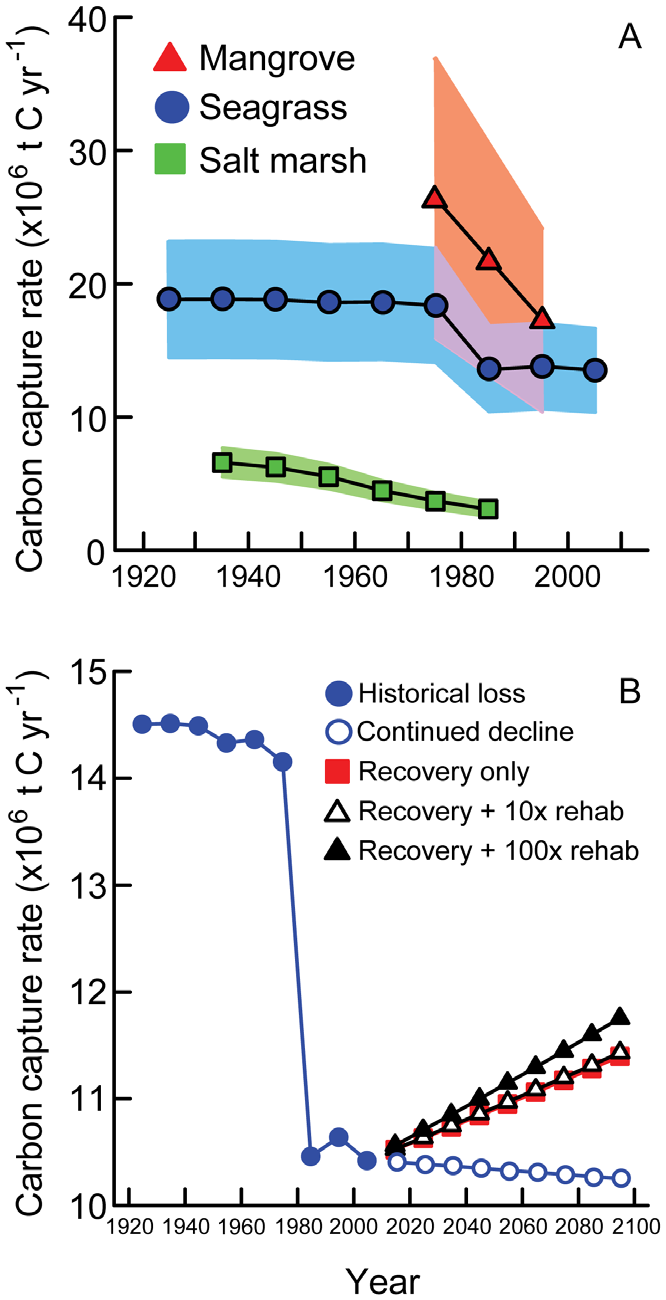
Historical and future carbon capture and storage rates (CCS) of coastal vegetation. (A) Extensive historical losses of seagrasses, mangroves, and salt marshes have reduced the CCS capacity of the coast. Points plotted represent the mean CCS for each habitat over time, and are bounded by lines of maximum and minimum rates of CCS published in the literature. Note that minimum rates for mangroves overlaps with the range of values for seagrass (depicted with purple shading). (B) Historical rates of CCS by seagrass are compared to rates under future scenarios of natural habitat recovery, as well as recovery combined with different intensities of restoration. Increasing restoration efforts to 100-times current levels will produce benefits to CCS that are similar to natural recovery alone. Rates of CCS following current trends in continued global seagrass decline are also plotted for reference. Data for calculating CCS rates were primarily sourced from [8], [9], [12], [13], [15], [16], [34] (also see Table 1).

Seagrasses, mangroves, and salt marshes have all experienced substantial declines in abundance that has reduced the global CCS achieved by these coastal habitats. The greatest changes have occurred within mangrove forests, where world-wide habitat losses exceeding 90,000 km^2^ since the 1970s have reduced their average global rate of CCS from ∼26.5×10^6^ t C yr^−1^ to ∼17.3×10^6^ t C yr^−1^ (Fig. 2A). Seagrass loss has occurred steadily since at least the early 1900s, but rates of loss peaked dramatically during the 1970s and 1980s, producing a rapid areal decline of over 44,000 km^2^ during this period alone. Concomitantly, rates of global CCS by seagrass declined by ∼4×10^6^ t C yr^−1^ to current average CCS of ∼16.7×10^6^ t C yr^−1^. Lastly, data available for salt marshes in the continental USA show a sustained rate of decline of ∼5% cover per decade since the 1930s, equating to losses approaching 20,000 km^2^ and a decline in average rates of CCS from ∼6.6×10^6^ t C yr^−1^ to ∼3.1×10^6^ t C yr^−1^.

### Improvements to global CCS

Using a global seagrass recovery rate of 1.08% per decade [16] and a conservative CCS rate of 83 t C km^−2^ yr^−1^ [9], natural seagrass recovery alone may produce global rates of CCS of ∼11.4×10^6^ t C yr^−1^ by the year 2100, an increase of ∼10% above current rates (Fig. 2B). Continuing along this trajectory, rates of CCS would reach 1920 levels (see ‘historical loss’ data: Fig. 2B) sometime around the year 2340. This timeframe may be shortened if rising CO_2_ increases seagrass productivity [25] and reduces covers of calcareous epiphytic algae that smother seagrasses [26].

Combining natural recovery with a global replication of current seagrass restoration efforts (4.59 km^2^) each decade until 2100 would improve rates of CCS by just 0.1% above benefits provided by natural recovery alone. Increasing seagrass restoration efforts 10-fold (45.94 km^2^ per decade) would provide a 0.9% improvement over natural recovery, but a 100-fold increase to what would likely require industrial-scale operations (459.4 km^2^ per decade) boosts CCS by a further 9.3%, resulting in rates of ∼11.8×10^6^ t C captured yr^−1^ (Fig. 2B). Such large-scale efforts would generate a return to 1920 levels of CCS around the year 2260, ∼80 years sooner than relying on natural recovery alone.

## Discussion

The link between rising concentrations of atmospheric CO_2_ and associated impacts of climate change has been argued to be one of the greatest challenges facing our understanding and management of the world's natural resources [27]. The sheer volume of anthropogenic carbon produced from fossil fuels and industry, ∼8,500×10^6^ t C yr^−1^ [9], [10], outweighs the CCS capacity of any natural habitat by at least an order of magnitude and immediately suggests that artificial methods of CCS are the only realistic CCS management option. However, artificial CCS methods, like most large-scale artificial management strategies [28], are currently burdened with uncertainty regarding their cost-effectiveness, long-term viability, and environmental dormancy. Natural CCS by aquatic and terrestrial vegetation offers a sustainable, low-risk, and potentially significant contribution toward managing the carbon problem, provided alarming historical trends in habitat degradation and loss can be slowed, arrested, or ideally, reversed.

Coastal vegetation such as mangroves, seagrasses, and salt marshes, have undergone extensive declines in abundance, distribution, and CCS over the past century. Based on the data available, we calculated a cumulative failure to capture at least 434×10^6^ t of carbon due to habitat loss since the 1920s. This value is certainly an underestimate, however, since data describing historical losses are typically limited (Fig. 2A), the baseline values used for calculations likely represent already impacted habitats (especially for mangroves where the earliest reliable data comes from the 1970s), and also because data for salt marshes, which have the greatest CCS potential of the three habitats examined [8], are restricted to the continental USA.

Can we rely on natural habitat recovery alone to regain lost CCS capacity among coastal habitats? Will coastal habitat restoration help improve global CCS? Using seagrasses as a model system, global recovery at a modest rate of 1.08% per decade over the next century may increase global CCS by seagrasses by ∼10% above current levels. However, this estimate depends on there being no further decline in net seagrass abundance, as well as the risky proposition that no major and as yet unforeseen future events will impact seagrass abundance during the decades to centuries needed for recovery [19]. Habitat restoration is a challenging but potentially powerful tool for further improving rates of CCS, yet the data suggest that efforts would need to be dramatically increased above current levels in order to contribute any significant effect beyond that gained through natural recovery alone. Industrial-scale restoration operations, in the order of 100s of kms restored per decade, may provide a substantial boost of ∼9% greater CCS above natural recovery alone (Fig. 2B). In isolation, such improvements to seagrass CCS would equate to the capture of ∼0.14% of predicted annual carbon emissions in 2100 (based on emission scenario A1B) [29]. While this proportion could certainly be improved by considering recovery and restoration of additional aquatic and terrestrial habitats, it still would not compensate for anthropogenic emissions [9], [10]. The cost of restoring such large amounts of seagrass could average ∼US$224 million yr^−1^ (based on restoring 459.4 km^2^ per decade), which may become cheaper if better techniques reduce costs from the current estimate of ∼US $48,700 ha^−1^, and if financial incentives can be provided through carbon trading schemes.

If such restoration is possible, maintaining abundant and optimally functioning coastal habitats may provide benefits against a changing climate that go beyond improved CCS. The effects of dense stands of terrestrial vegetation on local climatic conditions, such as reducing temperatures and desiccation stress, are well-known [30], [31]. Recent evidence suggests that such effects may represent disproportionately large buffers against forecast impacts of climate change both on land [32] and in the sea (e.g. kelp forests: Falkenberg, Russell and Connell unpubl. data). Thus, present-day investment in the maintenance and expansion of vegetation appears likely to not only improve global rates of CCS, but may also provide additional future rewards by lessening impacts of climate change.

Slowing or even reversing trends in net global habitat loss to improve the natural CCS capacity of the Earth is far easier said than done since CCS, and the benefits it may provide, is only one aspect of a complex issue. Often, the original reasons for habitat loss centre on direct tangible economic and social improvements. For example, one of the greatest threats to mangrove forests is the clear-felling of extensive areas to create space for commercial pond aquaculture of fish and crustaceans [15]. Such practices are most common in developing countries, particularly in SE Asia, and although mangrove removal radically alters the local ecology [12], the resulting land-use provides significant and much-needed socio-economic benefits. While coastal environments around the world have a long history of exploitation [18], it would ideally be managed to minimize long-term environmental impacts while still providing sustainable socio-economic rewards. Currently, such outcomes appear in the minority, yet there is encouraging evidence that it is achievable (e.g. sustainable rotating harvest of ∼1000 ha yr^−1^ of mangroves for wood since 1906: [12]).

Recognition of the value in reversing alarming trends of habitat loss is certainly not new, yet the importance of achieving such goals becomes clearer as we continue to learn about the numerous benefits that optimally functioning habitats can provide. CCS appears to be an increasingly valuable function of natural habitats, and habitat restoration may offer a solution to increase natural CCS at faster rates than through natural recovery alone. For coastal habitats such as seagrasses, it appears that increases in long-term CCS could be negligible unless restoration efforts can be increased to industrial-scale operations and/or restoration success rates can be improved through greater investment in research and methodology [33]. If such outcomes are beyond our technical expertise and political resolve, then questions must be asked about whether resources currently used for expensive and time-consuming restoration projects may instead be more wisely invested in arresting further habitat loss and encouraging natural recovery by mitigating pollutants and other impacts. While restoration even on a small scale certainly provides many benefits beyond CCS (e.g. habitat for other plants and animals, nutrient cycling, etc.), it appears that one of the most effective opportunities for mitigating climate effects is to reduce non-climate human impacts that are under local control, and thereby encourage natural habitat recovery.

## References

[1] Hoffert MI, Caldeira K, Benford G, Criswell DR, Green C (2002). Advanced technology paths to global climate stability: Energy for a greenhouse planet.. Science.

[2] Rogelj J, Nabel J, Chen C, Hare W, Markmann K (2010). Copenhagen accord pledges are paltry.. Nature.

[3] Wigley TML (2006). A combined mitigation/geoengineering aproach to climate stabilization.. Science.

[4] Walsh B (2009). Can geoengineering help slow global warming?.

[5] Chalmers H, Gibbins J (2010). Carbon capture and storage: the ten year challenge.. Proceedings of the Institute of Mechanical Engineering.

[6] Bachu S (2008). CO_2_ storage in geological media: Role, means, status and barriers to deployment.. Progress in Energy and Combustion Science.

[7] Lal R (2008). Carbon sequestration.. Philosophical Transactions of the Royal Society of London B.

[8] Laffoley DdA, Grimsditch G (2009). The Management of Natural Coastal Carbon Sinks.

[9] Pidgeon E, Laffoley DdA, Grimsditch G (2009). Carbon sequestration by coastal marine habitats: Important missing sinks.. The Management of Natural Coastal Carbon Sinks.

[10] Raupach MR, Marland G, Ciais P, Le Quere C, Canadell JG (2007). Global and regional drivers of accelerating CO_2_ emissions.. Proceedings of the National Academy of Sciences of the United States of America.

[11] Sabine CL, Feely RA, Gruber N, Key RM, Lee K (2004). The ocean sink for anthropogenic CO_2_.. Science.

[12] Alongi DM (2002). Present state and future of the world's mangrove forests.. Environmental Conservation.

[13] Duarte CM, Middelburg JJ, Caraco N (2005). Major role of marine vegetation on the oceanic carbon cycle.. Biogeosciences.

[14] Kennish MJ (2001). Coastal salt marsh systems in the U.S.: A review of anthropogenic impacts.. Journal of Coastal Research.

[15] Valiela I, Bowen JL, York JK (2001). Mangrove forests: One of the world's threatened major tropical environments.. Bioscience.

[16] Waycott M, Duarte CM, Carruthers TJB, Orth RJ, Dennison WC (2009). Accelerating loss of seagrasses across the globe threatens coastal ecosystems.. Proceedings of the National Academy of Sciences of the United States of America.

[17] Cebrián J (2002). Variability and control of carbon consumption, export, and accumulation in marine communties.. Limnology and Oceanography.

[18] Vitousek PM, Mooney HA, Lubchenco J, Melillo JM (1997). Human domination of Earth's ecosystems.. Science.

[19] Kirkman H, Kuo J (1990). Pattern and process in southern Western Australian seagrasses.. Aquatic Botany.

[20] Fonseca MS, Kenworthy WJ, Thayer GW (1998). Guidelines for the conservation and restoration of seagrasses in the United States and adjacent waters.

[21] Orth RJ, Carruthers TJB, Dennison WC, Duarte CM, Fourqurean JW (2006). A global crisis for seagrass ecosystems.. Bioscience.

[22] Orth RJ, Harwell MC, Inglis GJ, Larkum AWD, Orth RJ, Duarte CM (2006). Ecology of seagrass seeds and seagrass dispersal processes.. Seagrasses: Biology, Ecology and Conservation.

[23] Harley CDG, Hughes AR, Hultgren KM, Miner BG, Sorte CJB (2006). The impacts of climate change in coastal marine systems.. Ecology Letters.

[24] Green EP, Short FT (2003). World Atlas of Seagrasses.

[25] Palacios SL, Zimmerman RC (2007). Resonse of eelgrass *Zostera marina* to CO_2_ enrichment: possible impacts of climate change and potential for remediation of coastal habitats.. Marine Ecology Progress Series.

[26] Russell BD, Thompson J-AI, Falkenberg LJ, Connell SD (2009). Synergistic effects of climate change and local stressors: CO_2_ and nutrient-driven change in subtidal rocky habitats.. Global Change Biology.

[27] Kennedy D, Norman C (2005). What don't we know?. Science.

[28] Russell BD, Connell SD (2010). Honing the geoengineering strategy.. Science.

[29] Meehl GA, Stocker TF, Collins WD, Friedlingstein P, Gaye AT, Solomon S, Qin D, Manning M, Chen Z, Marquis M (2007). Global climate projections.. Climate Change 2007: The Physical Science Basis Contribution of Working Group I to the Fourth Assessment Report of the Intergovernmental Panel on Climate Change.

[30] Callaway RM (1995). Positive interactions among plants.. The Botanical Review.

[31] Goldenheim WM, Irving AD, Bertness MD (2008). Switching from negative to positive density-dependence among populations of a cobble beach plant.. Oecologia.

[32] McAlpine CA, Syktus J, Ryan JG, Deo RC, McKeon GM (2009). A continent under stress: interactions, feedbacks and risks associated with impact of modified land cover on Australia's climate.. Global Change Biology.

[33] Irving AD, Tanner JE, Seddon S, Miller D, Collings GJ (2010). Testing alternate ecological approaches to seagrass rehabilitation: links to life-history traits.. Journal of Applied Ecology.

[34] Watzin MC, Gosselink JG (1992). The fragile fringe: Coastal wetlands of the continental United States.

